# Changes in physical activity and sedentary time in United States adults in response to COVID-19

**DOI:** 10.1371/journal.pone.0273919

**Published:** 2022-09-09

**Authors:** Charles E. Matthews, Pedro Saint-Maurice, Janet E. Fulton, Shreya Patel, Erikka Loftfield, Joshua N. Sampson, Sarah K. Keadle, David Berrigan

**Affiliations:** 1 Metabolic Epidemiology Branch, Division of Cancer Epidemiology and Genetics, National Cancer Institute, Rockville, Maryland, United States of America; 2 Division of Nutrition, Physical Activity, and Obesity, National Center for Chronic Disease Prevention and Health Promotion, CDC, Atlanta, Georgia, United States of America; 3 Biostatistics Branch, Division of Cancer Epidemiology and Genetics, National Cancer Institute, Rockville, Maryland, United States of America; 4 Food and Drug Administration, Silver Spring, Maryland, United States of America; 5 Kinesiology and Public Health Department, California Polytechnic State University, San Luis Obispo, California, United States of America; 6 Health Behaviors Research Branch, Division of Cancer Control and Population Sciences, National Cancer Institute, Rockville, Maryland, United States of America; Public Health Agency of Canada, CANADA

## Abstract

Physical activity is associated lower risk for a broad range of non-communicable diseases and early mortality, and even small changes in daily activity levels could have a profound effect on public health at the population level. The COVID-19 pandemic reshaped daily life for United States (US) adults resulting in reductions in physical activity early in the pandemic, but its longer-term effects on daily activities are unknown. To examine the longer-term impact of the pandemic on daily activity levels, we conducted a nationwide longitudinal study of 1,635 adults (20–75 years) in AmeriSpeak. Previous-day recalls of time-use, sedentary time, and physical activity were completed on randomly selected days in Fall 2019 (pre-pandemic) and Fall 2020. Overall, US adults reported less time in transportation (-0.47 hrs/d), more total discretionary time (0.40 hrs/d), but no changes in total sedentary time (0.10 hrs/d) or leisure-time physical activity (-0.06 hrs/d). Women reported significantly less total activity (-0.36 hrs/d) and participants with children < 13 yrs reported more sedentary time (0.60 to 0.82 hrs/d) and less moderate-to-vigorous intensity activity (-0.84 to -0.72 hrs/d). Adults without children reported no changes in sedentary time (0.02 hrs/d) or moderate-vigorous intensity activity (-0.06 hrs/d). Adults who started working from home reported no changes in physical activity, but they were among the most sedentary and least active population groups at both timepoints. Our findings describe the complex inter-play between competing behaviors as time-use demands have changed in response to the pandemic, particularly for adults with younger children. Many US adults are likely to continue working from home; therefore, implementation of evidence-based approaches to increase physical activity and reduce sedentary time in this growing population subgroup appears warranted.

## Introduction

The COVID-19 pandemic dramatically reshaped daily life in the United States and worldwide [1]. Lockdowns early in the pandemic and ongoing community-based mitigation strategies have resulted in changes in how people spend their time in physically active and sedentary behaviors each day within occupational, transportation, educational, caring, and leisure and recreational domains. While initial pandemic lockdowns appeared to be associated with a significant reduction in daily physical activity [2–4], our understanding of longer-term effects of ongoing and renewed mitigation efforts is still evolving [5]. This could be an enduring public health concern because excessive sedentary time and inadequate moderate-to-vigorous intensity physical activity are associated with increased risk of early mortality, cardiometabolic disease, and certain cancers [6].

The initial United States lockdown (spring 2020) was associated with a 12% reduction in daily step counts among Fitbit users [2]. Furthermore, mobility patterns among Google users who shared location data also changed dramatically in this period. Time spent at workplaces, transit stations and retail centers [7] decreased substantially, whereas time spent at home and in parks and recreational spaces increased [7]. This raises the questions of whether the pandemic may have adverse long-term effects on daily activity levels or whether it may have *increased* participation in leisure-time activities (i.e., recreational and fitness related activities)—a potential benefit of the pandemic that could compensate for possible reductions in activity in other life domains. Additionally, rates of telecommuting increased dramatically [8, 9] and reductions in childcare support and school closures have reshaped family life dramatically, with particularly strong impacts on women in young families [1, 8]. For example, early in the pandemic women were shown to be spending more time on childcare and chores [1] and closures of school and childcare affected the work and homelife of parents more generally [8].

To date, most studies examining changes in physical activity and related behaviors were conducted early in the pandemic using convenience samples [2, 7] with cross-sectional or retrospective study designs [10–13]. Results from studies examining the impact of work status and changes in work location have been mixed suggesting either no changes in physical activity [14] or increased sedentary behavior [10] for those who started working from home. However, results from longitudinal and more representative population-based samples in US adults later in the pandemic are few. Thus, the longer-term effects of the pandemic on physical activity and sedentary time have not been established. Additionally, there has been only limited inquiry into how extended changes in home-life (e.g., childcare), work status and location (e.g., working from home), and daily transportation may have altered daily physical activity patterns. To address these gaps we carried out a longitudinal study using nationwide survey data collected prior to the pandemic in the Fall of 2019 [15, 16] and new data collected in the same individuals in Fall 2020 using a validated previous-day recall to assess domain-specific changes in daily physical activities and sedentary behaviors [17, 18]. Such data are informative for identifying post-COVID-19 targets for public health action aimed at reducing high levels of sedentary time [16] and supporting the accumulation of recommended amounts of moderate-to-vigorous intensity physical activity [6].

## Materials and methods

### Study population and design

Participants were from AmeriSpeak, a probability-based panel designed to represent the US population [19, 20]. The National Opinion Research Center (NORC) at the University of Chicago developed, maintains, and gathers IRB approved informed consent for AmeriSpeak when participants enroll in the panel, which was obtained in-person, by mail, or through a website. A general sample of the panel 20–75 years of age who could complete online surveys were invited to participate. Pre-pandemic data was collected in Fall 2019 (Oct 16 to Nov 11, 2019) and in the Fall of 2020 (Nov 3 to Nov 15, 2020). All participants in the 2019 survey were invited to participate in 2020.

Panelists were sent e-invitations to complete a short online-survey and unannounced 24-hour recalls on randomly selected days. In Fall 2019 participants were asked to complete two recalls in a 1–2-week period, and in 2020 they were asked to complete one recall. Recalls could only be completed on the randomly targeted recall day and no make-up recalls on later days were attempted. The online survey included questions about self-rated health, physical activity, height, weight, and current occupational status and location of work. Additional demographic information including self-reported race and ethnicity was collected at enrollment into the AmeriSpeak panel. Participants received $15/recall. Data from this study can be found in the S1 Data.

### Development of survey sample weights

The AmeriSpeak panel is based on a stratified two-stage sampling design. Primary sampling units in the first stage are National Frame Areas and the secondary sampling units are defined from Census tracts or block groups. To develop our sample weights, we started with the final weights calculated for each AmeriSpeak panelist in our longitudinal sample and for each recall we calculated study-specific weights that also adjusted for selection probabilities from the panel, non-response in our study, and population coverage. Final weights are further adjusted (i.e., raked) to external population totals with respect to age, sex, education, race and Hispanic ethnicity, housing tenure, telephone status, and Census Division derived from the Current Population Survey [19]. Sampling weights for each day of the week were calculated separately (e.g., the participants who completed the recall on a Monday are weighted to represent the US population) and we then further normalized the weights, so each day of the week contributed equally (i.e., after weighting, each day of the week has an equal number of recalls).

### Activities completed over time in 24-hours (ACT24)

Recalls were completed online using the self-administered ACT24 previous-day recall [17]. To complete a recall (via smartphone, tablet, or computer) participants reported how they spent their time in-bed/sleeping, being physically active, and in sedentary behaviors on the previous day (midnight-midnight) by selecting from 170+ individual activities organized in 14 major categories (S1 Table). Reportable physically active behaviors included light (non-sedentary, < 3.0 METs), moderate (3–5.9 METs) and vigorous intensity (≥ 6.0 METs) activities. To minimize respondent burden and to simplify scoring, participants are asked to report only their primary activities, although the system will allow overlapping activities to be entered. After selecting an activity, follow-up questions assessed the duration of the activity, body position, and other details. Time-use in specific life domains was classified to be consistent with the American Time-use Survey (ATUS) [21] (S2 Table). Sedentary behaviors were defined as sitting/reclining with little energy expenditure (typically ≤ 1.5 METs) while not in-bed/sleeping. We labeled time in-bed, and time sedentary and physically active as “daily behaviors” to distinguish them from the distinct behaviors reported in the major time-use domains. The “waking day” was defined as time out of bed for the day. During data collection a “provisionally valid” recall was defined as one in which participants reported at least 2 activities and 22 hours of information. After field work was complete additional quality control checks were applied and for recalls with more than one activity reported at the same time, the most active behaviors for the overlapping time were selected, and time reported in each behavior was recalculated. An earlier version of ACT24 was found to be accurate in estimating physically active and sedentary time at the population level in comparison to activPAL [17] in middle-aged and older adults, and similar recall-based methods have been found to provide useful estimates of domain-specific behaviors [22, 23]. In 47 adults 20–73 years of age the current version of ACT24 provided accurate estimates of mean time in sedentary behavior and physical activity compared to activPAL (sedentary: 9.1 (SD = 2.3) vs. 9.3 (2.1) hrs/d; activity: 6.4 (SD = 2.1) vs. 6.3 (1.9) hrs/d), and a relatively high correlation between measures (Spearman rho = 0.61, sedentary; rho = 0.65, activity; unpublished observations). ACT24 is freely available for researchers to use (https://dceg.cancer.gov/research/how-we-study/exposure-assessment/physical-activities-completed-over-time-24-hours-act-24).

### Statistical analysis

We described the characteristics for participants who completed recalls in 2019 and 2020. We tabulated the actual unweighted number of participants and the weighted population percentages in each demographic category. For 2019, we calculated the mean of each time variable (e.g., number of hours spent in sedentary activity) using observations from both 2019 recalls, and for 2020, we calculated the mean of each time variable using observations from the sole recall. Using survey methods and the weights described above, we obtained unbiased estimates of population averages and robust standard errors. For estimating the differences between 2019 and 2020, we regressed each time variable on study year using observations from available recalls. We repeated the regression in strata defined by gender (female, male), age (20–39, 40–64, 65–76 yrs), family structure at home (no children <18 and children <5, 6–12, 13–17 yrs), and current work from home status (yes, all or some of the time, not working from home, unemployed, retired/disabled). All analyses were performed in SAS 9.4 using survey procedures (PROC SURVEYREG, PROC SURVEYMEANS, and PROC SURVEYFREQ) to account for the complex sampling design. Statistical significance of two-sided tests was defined as p-value < 0.05 and did not account for multiple testing.

## Results

### Enrollment into study

In 2019, 19% of panelists (2,877 of 15,153 invited) completed the short survey and a provisionally valid recall. Participants from 2019 who were still active in AmeriSpeak in 2020 were invited to participate in 2020, and 69% (1,788 of 2,574 invited) completed a second survey and an additional recall. After additional ACT24 quality control processing was completed, 1,635 of the 1,788 (91%) longitudinal participants remained eligible for analysis. Comparison with demographic characteristics of the US population in the Current Population Survey revealed few differences in our weighted demographic data from the longitudinal sample with provisionally valid recalls, except for an overrepresentation of participants with an annual income of less than $50K and an underrepresentation of participants reporting $150K or more (S3 Table). The descriptive characteristics of our final analytic sample are provided in Table 1.

**Table 1:** Descriptive characteristics of the analytic sample^a^.

	All Participants	Male	Female
	(n = 1,635)	(n = 913)	(n = 722)
Characteristic	Frequency	Frequency	Frequency
	(weighted %)	(weighted %)	(weighted %)
**Age (years)**			
20–39	654 (39.1)	312 (32.4)	342 (45.7)
40–64	758 (44.7)	462 (50.3)	296 (39.3)
65–76	223 (16.1)	139 (17.3)	84 (15.0)
**Race and ethnicity**			
White, non-Hispanic	1171(62.8)	685 (68.5)	486 (57.4)
Black, non-Hispanic	146(10.6)	58 (9.6)	88 (11.9)
Hispanic	181(16.9)	79 (12.0)	102 (21.7)
Additional groups^b^	137(9.7)	91 (10.4)	46 (9.0)
**Educational Attainment**			
High School or less	202(32.5)	110 (32.7)	92 (32.3)
Some college/Assoc. Degree	599(28.8)	303 (26.4)	296 (31.0)
Bachelor’s Degree	480(21.4)	289 (23.7)	191 (19.4)
Graduate Degree	354(17.4)	211 (17.2)	143 (17.5)
**Household Income ($)**			
< 50,000	560 (40.6)	269 (37.2)	291 (43.8)
50,000–99,000	603 (34.5)	352 (36.6)	251 (32.6)
100,000–149,000	290 (15.3)	169 (14.8)	121 (15.7)
150,000+	182 (9.6)	123 (11.4)	59 (7.9)
**Occupational Status**			
Working for pay	1195 (66.7)	690 (71.0)	505 (62.7)
Unemployed	181 (13.4)	64 (9.0)	117 (17.7)
Retired	189 (14.6)	115 (14.0)	74(15.2)
Disabled	70 (5.2)	44 (6.0)	26 (4.4)
**Updated Occupational Status**			
Working for pay	1097 (61.9)	629 (65.6)	468 (58.2)
Unemployed	133 (8.9)	76 (9.6)	57 (8.3)
Retired	257 (18.5)	132 (16.3)	125 (20.5)
Disabled	46 (2.8)	30 (3.8)	16 (1.9)
Missing	102 (7.9)	46 (4.7)	56 (11.0)
**Change in work location**			
Yes	465 (25.1)	271 (27.6)	194 (22.7)
No	631 (36.7)	357 (37.9)	274 (35.5)
Unemployed	157 (11.3)	89 (10.5)	68 (12.0)
Retired/Disabled	298 (21.1)	159 (19.9)	139 (22.3)
Missing/Unknown	84 (5.8)	37 (1.0)	47 (7.5)
**Working from home**			
Yes, all or some of the time	400 (20.8)	230 (22.0)	170 (19.6)
Not working from home	697 (41.0)	399 (43.6)	298 (38.6)
Unemployed	157 (11.3)	89 (10.5)	68 (12.0)
Retired/Disabled	298 (21.1)	159 (19.9)	139 (22.3)
Missing/Unknown	83 (5.7)	36 (4.0)	47 (7.5)
**Children in household**			
No children <18 years old	1138 (69.1)	671 (73.6)	467 (64.8)
<5 years old	176 (10.5)	83 (8.0)	93 (12.9)
6–12 years old	306 (18.8)	140 (14.1)	166 (23.3)
13–17 years old	231 (15.1)	120 (14.3)	111 (15.9)
**Body Mass Index (kg/m2)**			
< 25	478(27.9)	232 (23.6)	246 (32.0)
25–29.9	524 (32.6)	344 (37.0)	180 (28.3)
30+	588 (37.0)	313 (37.0)	275 (37.1)
Missing	45 (2.5)	24 (2.3)	21 (2.6)
**Aerobic Physical Activity**			
Inactive	271 (17.4)	145 (17.5)	126 (17.4)
Insufficiently Active	480 (28.6)	257 (27.1)	223 (30.0)
Sufficiently Active	340 (21.8)	186 (19.7)	154 (23.7)
Highly Active	532 (31.3)	320 (35.0)	212 (27.7)
Missing	12 (0.9)	5 (0.6)	7 (1.2)
**Region**			
Northeast	252 (17.6)	147 (18.9)	105 (16.3)
Midwest	452 (20.1)	242 (20.2)	210 (20.0)
South	520 (38.0)	275 (35.1)	245 (40.9)
West	411 (24.3)	249 (25.9)	162 (22.8)

^a^Analytic sample after exclusions following ACT24 quality control procedures

^b^Additional groups include, individuals self-identifying as non-Hispanic Asian, Other, or being from more than one race or ethnic group

### Longitudinal changes in the overall sample: Time in-bed, sedentary time, and physical activity

**Fig 1:**
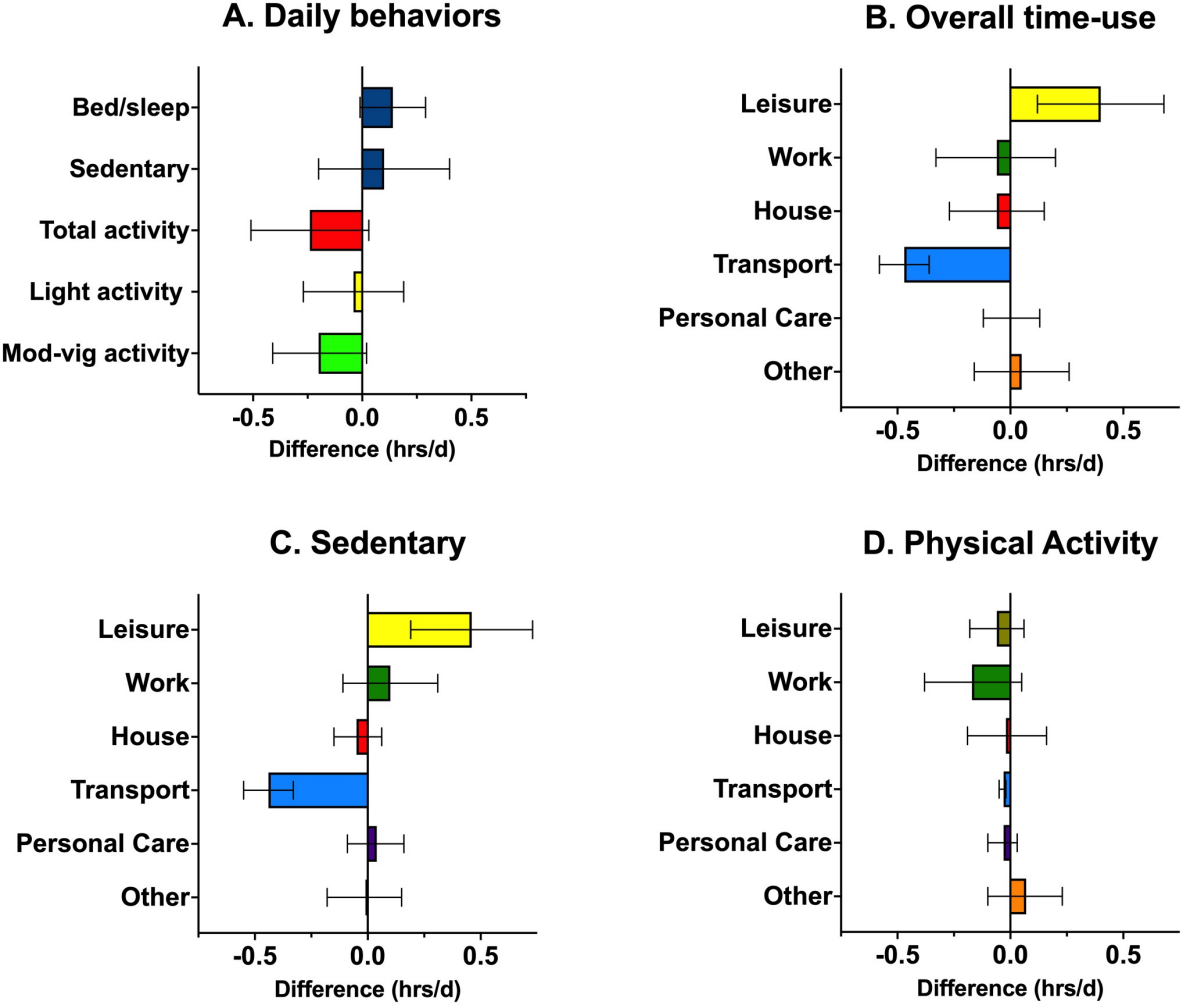
Mean differences (95%CIs) between Fall 2019 and Fall 2020 in daily behaviors, overall time-use in the waking day, sedentary behavior, and physical activity. The Other category is composed of Categories with a low prevalence of reporting (Church or spiritual pursuits; Volunteer; School/education; Private time; Unreported (gap) time).

We observed small non-significant increases (p > 0.05) in time reported in-bed/sleeping (0.14 hrs/d) and sedentary time (0.10 hrs/d) in 2020 and corresponding non-significant reductions in total (-0.24 hrs/d) and moderate-to-vigorous intensity activity (-0.20 hrs/d; Fig 1, Panel A; Table 2). Significant domain specific differences (p < 0.05) were observed for overall time use (i.e., combined sedentary and active) with an increase in overall leisure time (0.40 hrs/d) in 2020, and reductions in total transportation in 2020 (-0.47 hrs/d; Fig 1, Panel B; Table 3). The apparent reallocation of time from transport to discretionary leisure-time in 2020 co-occurred with an increase in time spent in sedentary leisure pursuits (+0.46 hrs/d; Fig 1, Panel C; Table 3), but there was no change in physical activity during leisure-time (-0.06 hrs/d; Fig 1, Panel D; Table 3). Further analysis indicated that increased sedentary leisure time was driven by increased screen time (0.45 hrs/d). Significant reductions in sedentary (-0.44 hrs/d; Panel C; Table 3) and active transport (-0.03 hrs/d; Fig 1, Panel D; Table 3) were also observed.

**Table 2:** Difference in time spent (hours/day) in daily behaviors pre-pandemic in Fall 2019 and Fall 2020, by sex and age-group—US adults.

Category	Metric	Mean 2019	Mean 2020	Difference (Δ)	P
**Overall sample** (n = 1,635)	Bed/sleep	8.07	8.20	0.14	0.07
	Sedentary	9.69	9.79	0.10	0.51
	Total Physical Activity	6.25	6.01	-0.24	0.09
	Light	3.91	3.87	-0.04	0.72
	Moderate-vigorous	2.34	2.15	-0.20	0.08
**Men** (n = 913)	Bed/sleep	7.89	7.99	0.11	0.24
	Sedentary	10.11	10.11	0.01	0.98
	Total Physical Activity	6.01	5.91	-0.11	0.64
	Light	3.39	3.43	0.04	0.85
	Moderate-vigorous	2.62	2.47	-0.15	0.37
**Women** (n = 722)	Bed/sleep	8.24	8.41	0.17	0.21
	Sedentary	9.28	9.48	0.20	0.26
	Total Physical Activity	**6.49**	**6.12**	**-0.36**	**0.03**
	Light	4.42	4.29	-0.13	0.33
	Moderate-vigorous	2.07	1.83	-0.24	0.07
**Age 20–39** (n = 654)	Bed/sleep	8.35	8.38	0.03	0.80
	Sedentary	9.21	9.37	0.16	0.54
	Total Physical Activity	6.45	6.26	-0.19	0.44
	Light	3.89	3.99	0.10	0.64
	Moderate-vigorous	2.56	2.27	-0.29	0.21
**Age 40–64** (n = 758)	Bed/sleep	**7.86**	**8.12**	**0.25**	**0.05**
	Sedentary	9.92	9.83	-0.09	0.69
	Total Physical Activity	6.23	6.07	-0.16	0.46
	Light	3.93	3.89	-0.04	0.86
	Moderate-vigorous	2.30	2.17	-0.12	0.47
**Age 65–76** (n = 223)	Bed/sleep	7.92	8.02	0.10	0.60
	Sedentary	10.35	10.71	0.37	0.36
	Total Physical Activity	5.74	5.28	-0.46	0.21
	Light	3.89	3.51	-0.38	0.10
	Moderate-vigorous	1.85	1.77	-0.09	0.76

**Table 3:** Changes (Δ) in total time-use, sedentary time, and physical activity pre-pandemic in Fall 2019 and Fall 2020, overall and by sex and age-group—US adults.

		Time-use Total (hrs/d)			Sedentary Time (hrs/d)			Physical Activity Duration (hrs/d)		
	Domain	Mean2019	Δ	p	Mean2019	Δ	p	Mean2019	Δ	p
**Overall sample** (N = 1,635)	Leisure	**5.46**	**0.40**	**0.01**	**4.51**	**0.46**	**< .01**	0.96	-0.06	0.34
	Work	3.70	-0.06	0.63	1.91	0.10	0.33	1.79	-0.17	0.13
	House	2.96	-0.06	0.55	0.50	-0.05	0.39	2.46	-0.02	0.87
	Transport	**1.19**	**-0.47**	**< .01**	**1.13**	**-0.44**	**< .01**	**0.06**	**-0.03**	**< .01**
	Personal Care	1.79	0.00	0.96	0.97	0.04	0.56	0.82	-0.03	0.32
	Other	0.83	0.05	0.61	0.67	-0.01	0.87	0.16	0.07	0.42
**Men** (n = 913)	Leisure	**5.81**	**0.48**	**0.02**	**4.83**	**0.43**	**0.04**	0.97	0.04	0.71
	Work	4.20	0.02	0.92	2.04	0.21	0.11	2.16	-0.19	0.25
	House	2.36	-0.18	0.12	**0.39**	**-0.10**	**0.05**	1.97	-0.08	0.39
	Transport	**1.23**	**-0.49**	**< .01**	**1.16**	**-0.46**	**< .01**	**0.07**	**-0.04**	**0.01**
	Personal Care	1.75	-0.03	0.64	1.01	-0.01	0.90	0.74	-0.03	0.53
	Other	0.77	0.11	0.46	0.67	-0.08	0.30	0.10	0.19	0.18
**Women** (n = 722)	Leisure	5.13	0.34	0.06	**4.18**	**0.49**	**0.01**	**0.94**	**-0.15**	**0.04**
	Work	3.20	-0.14	0.39	1.78	0.00	0.98	1.42	-0.13	0.28
	House	3.55	0.04	0.77	0.61	0.00	0.99	2.95	0.04	0.74
	Transport	**1.16**	**-0.44**	**< .01**	**1.11**	**-0.42**	**< .01**	**0.05**	**-0.02**	**< .01**
	Personal Care	1.84	0.04	0.67	0.94	0.08	0.43	0.90	-0.04	0.44
	Other	0.89	0.00	0.98	0.67	0.05	0.68	0.22	-0.05	0.42
**Age 20–39** (n = 654)	Leisure	4.51	0.51	0.08	**3.59**	**0.54**	**0.05**	0.92	-0.03	0.71
	Work	4.40	-0.34	0.11	2.20	0.05	0.77	**2.20**	**-0.39**	**0.03**
	House	2.90	0.09	0.56	0.60	-0.04	0.60	2.30	0.13	0.35
	Transport	**1.17**	**-0.47**	**< .01**	**1.09**	**-0.42**	**< .01**	**0.08**	**-0.06**	**< .01**
	Personal Care	1.72	-0.03	0.79	0.92	-0.09	0.32	0.80	0.06	0.42
	Other	0.96	0.21	0.24	0.81	0.11	0.47	0.15	0.10	0.17
**Age 40–64** (n = 758)	Leisure	5.66	-0.02	0.92	4.74	0.07	0.72	0.92	-0.10	0.27
	Work	3.84	0.43	0.07	2.03	0.32	0.08	1.81	0.11	0.49
	House	2.90	-0.24	0.10	0.43	-0.07	0.42	2.46	-0.17	0.17
	Transport	**1.27**	**-0.46**	**< .01**	**1.22**	**-0.44**	**< .01**	0.05	-0.02	0.15
	Personal Care	1.76	0.01	0.96	0.96	0.08	0.38	**0.80**	**-0.08**	**0.02**
	Other	0.72	0.04	0.81	0.55	-0.06	0.47	0.17	0.10	0.55
**Age 65–76** (n = 223)	Leisure	**7.62**	**0.93**	**< .01**	**6.44**	**0.98**	**< .01**	1.18	-0.05	0.78
	Work	1.11	-0.25	0.23	0.62	-0.14	0.39	0.48	-0.12	0.36
	House	3.37	-0.02	0.95	0.42	0.02	0.85	2.94	-0.03	0.92
	Transport	**1.00**	**-0.46**	**< .01**	**0.98**	**-0.46**	**< .01**	0.03	0.00	0.79
	Personal Care	2.13	0.01	0.96	1.19	0.17	0.22	**0.93**	**-0.16**	**< .01**
	Other	0.86	-0.30	0.09	0.69	-0.20	0.24	**0.17**	**-0.10**	**0.04**

### Longitudinal changes by sex, age, and race-ethnicity

There were no significant changes in time reported in the daily behaviors pre-pandemic (2019) to 2020 in men, but women reported significantly less total physical activity (-0.36 hrs/d, Table 2). Men reported a significant increase in total leisure-time (0.48 hrs/d), but women did not (0.34 hrs/d). Both sexes reported similar reductions in total transportation time in 2020 (Table 3). Both men and women appeared to use time saved in transportation in sedentary rather than physically active pursuits. Further analysis indicated sedentary screen time during leisure was significantly greater in 2020 in women (0.48 hrs/d) and men (0.44 hrs/d). Men also reported less sedentary housework in 2020 (Table 3).

Changes in behavior differed by age. There was a significant increase in time reported in-bed/sleeping in 40–64-year-olds (0.25 hrs/d), but no changes in this metric in the other age-groups (Table 2). Adults aged 40–64 years also reported less time in transport in 2020 (-0.46 hrs/d) but this time savings appeared to be reallocated work (0.43 hrs/d) and time in-bed/sleeping, rather than overall leisure-time (-0.02 hrs/d; Table 3). Adults aged 20–39 yrs reported significant reductions in total transportation (-0.47 hrs/d), increases in sedentary leisure-time (0.54 hrs/d), reductions in work related activity (-0.39 hrs/d), and less active transport (-0.06; Table 3). Older adults (65–76 yrs) reported less time in transport overall (-0.46 hrs/d), more total time in leisure (0.93 hrs/d), and small reductions in physically active personal care (-0.16 hrs/d) and other activities (-0.10 hrs/d; Table 3). Results for adults identifying themselves in the major race and ethnic groups were broadly like differences noted above for reduced transportation, although smaller sample sizes reduced statistical power (S4 Table). Black adults reported increased in time in-bed/sleeping (0.82 hrs/d), but no increase in overall leisure-time (S4 Table).

### Longitudinal changes by family structure: Children in the household

**Fig 2:**
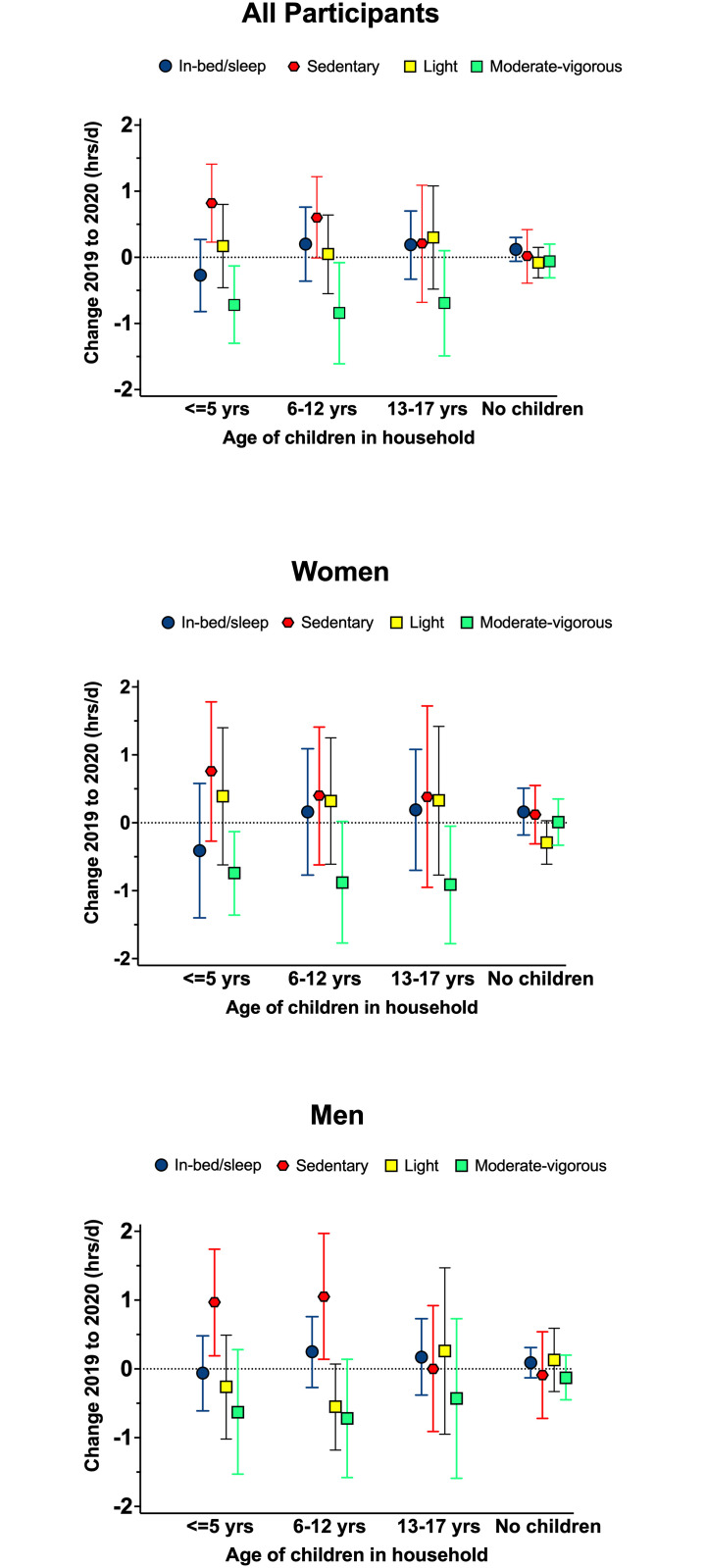
Mean differences (95%CIs) between pre-pandemic in Fall 2019 and Fall 2020, in daily behaviors in all participants and men and women, by the presence of children in the household.

Among participants with younger children (≤ 5, 6–12 yrs), we observed substantial increases in total sedentary time (0.60 to 0.82 hrs/d) and reductions in moderate-to-vigorous intensity physical activity (-0.84 to -0.72 hrs/d; Fig 2). Women with children 13–17 yrs also reported significantly less moderate-to-vigorous intensity physical activity (-0.91 hrs/d) in 2020. Among participants in the overall sample with no children < 18 yrs in their households, no changes in these behaviors were noted (Fig 2). Evaluation of changes in behavior by domain among men with younger children (≤ 5, 6–12 yrs) suggested that increases in total sedentary time were largely driven by increases in sedentary time at work and during leisure, while reductions in physical activity occurred in the work and household domains (S5 and S6 Tables). Women with children ≤ 5 yrs reported a non-significant (p > 0.05) increase in sedentary behavior across most domains other than transportation, while women with older children primarily reported increases in sedentary leisure-time. Reported reductions in physical activity among women with children ≤ 5yrs were largely driven by reductions in work related activity (S5 and S6 Tables).

### Longitudinal changes by current occupational status and working location

To evaluate the impact of changes in occupational status and work location during the pandemic we examined updated occupational information from our 2020 survey. Those who shifted to working from home “all of the time” or “some of the time” during the pandemic reported significantly more time in-bed/sleeping (0.40 hrs/d), while those who did not work from home or were unemployed or retired/disabled did not. Adults who reported being unemployed in 2020 reported significantly less moderate-to-vigorous intensity physical activity (-0.91 hrs/d; Table 4). Significant reductions in total time spent in transportation were reported in all occupational groups, but significant increases in total leisure-time were noted only for the unemployed and retired/disabled groups, groups who also reported significant reductions in work related physical activity (Table 4). Among those who were working during the pandemic, adults who worked from home reported a significant increase in sedentary work (0.54 hrs/d) which was offset by reductions in sedentary transportation (-0.72 hrs/d) and other sedentary behaviors (-0.41 hrs/d). Adults who were working outside the home reported a significant increase in total time working (0.37 hrs/d), and significant reductions in sedentary household activity (-0.13 hrs/d) and transportation (-0.32 hrs/d).

**Table 4:** Changes (Δ) in daily behaviors and time-use (hrs/d) between 2019 and 2020, by occupational and work from home status in 2020—US adult.

	Work from home			Not working from home			Unemployed			Retired/Disabled		
	(n = 400)			(n = 697)			(n = 157)			(n = 298)		
	Mean 2019	Δ	p	Mean 2019	Δ	p	Mean 2019	Δ	p	Mean 2019	Δ	p
**Daily behaviors**												
** ** In-bed/sleep	**7.96**	**0.40**	**0.01**	7.89	0.21	0.08	8.46	-0.08	0.79	8.31	-0.13	0.58
Sedentary	10.96	-0.36	0.21	9.13	-0.13	0.53	9.25	0.48	0.47	10.05	0.48	0.22
Total Physical Activity	5.09	-0.05	0.84	6.99	-0.08	0.69	6.30	-0.40	0.57	5.65	-0.35	0.27
Light	3.71	-0.24	0.23	3.86	0.08	0.66	4.07	0.51	0.44	3.92	-0.26	0.21
Moderate-vigorous	1.39	0.19	0.33	3.13	-0.17	0.51	**2.23**	**-0.91**	**0.01**	1.72	-0.09	0.67
**Total time-use**												
Leisure	4.40	0.28	0.26	4.83	-0.05	0.81	**5.55**	**1.16**	**0.03**	**7.76**	**0.77**	**0.01**
Work	5.36	0.37	0.28	**4.96**	**0.37**	**0.05**	**3.00**	**-1.49**	**< .01**	**0.56**	**-0.35**	**0.03**
House	2.26	0.28	0.13	2.63	-0.18	0.27	2.77	0.19	0.44	3.81	-0.10	0.69
Transport	**1.44**	**-0.81**	**< .01**	**1.28**	**-0.33**	**< .01**	**1.22**	**-0.60**	**0.01**	**0.83**	**-0.32**	**0.01**
Personal Care	1.75	-0.06	0.46	1.63	0.01	0.94	2.03	-0.13	0.57	2.01	0.11	0.46
Other	**0.84**	**-0.46**	**< .01**	0.79	-0.03	0.78	0.98	0.95	0.15	0.73	0.01	0.93
**Sedentary time-use**												
Leisure	3.56	0.20	0.34	3.92	0.09	0.59	**4.53**	**1.43**	**0.01**	**6.62**	**0.69**	**0.04**
Work	**4.00**	**0.54**	**0.05**	2.03	0.19	0.19	1.27	-0.37	0.15	0.30	-0.14	0.29
House	0.43	0.01	0.92	**0.48**	**-0.13**	**0.04**	0.39	0.00	0.98	0.54	0.04	0.78
Transport	**1.32**	**-0.72**	**< .01**	**1.23**	**-0.32**	**< .01**	**1.14**	**-0.53**	**0.01**	**0.80**	**-0.32**	**0.01**
Personal Care	0.96	0.02	0.69	0.87	0.04	0.54	1.07	-0.29	0.18	1.17	0.19	0.22
Other	**0.69**	**-0.41**	**< .01**	0.60	0.00	1.00	0.85	0.24	0.28	0.61	0.03	0.84
**Active time-use**												
Leisure	0.84	0.07	0.49	0.90	-0.14	0.22	1.03	-0.27	0.27	1.14	0.09	0.61
Work	1.36	-0.17	0.54	2.93	0.18	0.34	**1.73**	**-1.12**	**< .01**	**0.25**	**-0.22**	**0.01**
House	1.82	0.27	0.09	2.16	-0.05	0.72	2.39	0.19	0.44	3.26	-0.13	0.63
Transport	**0.12**	**-0.09**	**< .01**	0.05	-0.01	0.49	**0.08**	**-0.07**	**< .01**	0.03	0.00	0.82
Personal Care	0.79	-0.09	0.10	0.77	-0.04	0.35	0.96	0.16	0.47	0.85	-0.08	0.14
Other	0.15	-0.04	0.55	0.19	-0.03	0.67	0.12	-0.02	0.60	0.13	0.71	0.28

Although adults who started working from home during the pandemic did not report dramatic changes in physical activity or sedentary behavior, they reported the most sedentary time and least moderate-to-vigorous intensity activity of these occupational groups in 2019 (Table 4). In 2020, their patterns of sedentary behavior and physical activity were more like retired and disabled adults (S1 Fig).

## Discussion

In this longitudinal survey conducted in a nationwide sample before and during the COVID-19 pandemic (Fall 2020), US adults overall reported spending 0.47 hrs/d less total time in transportation and 0.40 hrs/d more in overall leisure time, but they did not report spending this newfound discretionary time in physically active exercise or recreational pursuits. While we found no major changes in levels of physical activity or sedentary behavior in the overall population, suggesting no change from pre-pandemic levels, several population subgroups did report significant changes that warrant attention. Women reported significantly less total physical activity during the pandemic, and men and women with younger children reported substantial increases in sedentary time and reductions in moderate-to-vigorous intensity activity. In contrast, adults who started working from home reported no major changes in sedentary time or physical activity, but this group was among the most sedentary and least active segment of the population both before and after the pandemic. In the following sections we expand on each of these findings, consider their implications, and discuss the strengths and limitations of this research.

To our knowledge this is the first detailed longitudinal analysis of changes in physical activity and sedentary behavior in a population-based sample of US adults well after the initial lockdowns arising from the COVID-19 pandemic. In the Fall of 2020 (November), the US was largely back “open” after the initial lockdowns, with the New York Times reporting that 31 states were “Open with some limits” or had “No restrictions”, 13 states were “Mostly open” and only one state was “Partly open” [24]. Never-the-less, during this period US adults reported spending less time engaged in transportation, a change that was associated with an increase in leisure time. In the US, transportation related to commuting and doing errands is largely motorized and sedentary, and US adults on average opted to exchange their time saved in daily transportation for increased time in sedentary leisure pursuits (0.46 hrs/d) rather than in exercise or fitness related activities (-0.06 hrs/d). These findings are broadly consistent with recent national data. The US Bureau of Travel Statistics report more people staying home and fewer trips taken in 2020 compared to 2019 [25]. The American Time Use Survey examined differences in overall time use between May and December of 2019 and the same months in 2020, and reported an average decrease of 0.43 hrs/d in transportation, an increase of 0.53 hrs/d in leisure-time, but little or no change in exercise and sports participation (0.05 hrs/d) [9]. Time use data from the United Kingdom (UK) has shown relatively large reductions in time working away from home (-1.3 hrs/d) in May/June of 2020 with partial rebound by August 2020 in comparison to 2016 data; changes that likely affected transportation [26]. Our findings are also consistent with changes in population-level mobility in the US between pre-pandemic levels and the period in which our survey was conducted (November 3–15, 2020). These changes indicate reductions in time spent in retail locations (-17%), transit stations (-34%) and workplaces (-26%) and more time in residential locations (+9%) [7].

Some, largely but not entirely, cross-sectional studies have indicated more dramatic changes in time use at specific times in the pandemic [5] and significant differences between men and women in time spent on necessities and household activities [1]. However, these studies were not optimized to assess levels of physical activity and sedentary time, a strength of our study since even small changes in daily physical activity can have a large public health impact at the population level [27]. Additionally, cross-sectional surveys, have more limited capacity to quantify the actual magnitude of changes in behavior compared to longitudinal study designs.

The time-use changes described above were the predominant patterns we observed in most population subgroups, although there were some exceptions. Adults aged 40–64 years reported spending less time in transportation, but also more time in-bed/sleeping (0.25 hrs/d) and more time working (0.43 hrs/d) rather than more leisure-time. Overall, women reported spending 0.36 hrs/d less in total physical activity later in the pandemic and this change appeared to be driven by the subgroup of women with likely demands associated with having children (< 18 yrs) in the household. This finding is broadly consistent with results from Curtis and colleagues [28] who reported significantly lower physical activity levels as measured by an accelerometer during the initial lockdowns in Australia. Recent ATUS data indicates that times spent in secondary childcare activities increased by about an hour per day in 2020 for both men and women with children under 13 years of age in the household [9]. In the present study the large reductions in moderate-to-vigorous intensity physical activity observed in adults with young children in the household are particularly worrisome because higher intensity activities such as these are recommended for better physical and mental health [29]. As the current pandemic continues, and in preparation for possible future pandemics, adults with young children, especially mothers [1] appear to be particularly vulnerable to reductions in activity levels potentially associated with increased educational, household chore and childcare demands. If pandemic conditions allow (e.g., low local rates of virus transmission and hospitalizations), supports that allow initiating or maintaining healthful physical activity levels may be needed. Implementing effective COVID-19 prevention strategies within schools and early childhood education programs [30] can help keep these facilities open for in-person learning while enabling parents, guardians, and caregivers to continue to work [31–33] and be in a better position to maintain healthful activity levels.

Changes in the location of the workplace, or working from home, may be one of the most enduring effects of the pandemic. Prior to the pandemic (2019), 22% of working adults in the US reported working from home, but during the pandemic (2020) this proportion nearly doubled, increasing to 42% [9]. Two cross-sectional studies that assessed physical activity and sedentary behavior only after the pandemic onset reported that Japanese [34] and US adults [10] who started working from home were less active and more sedentary than their counterparts who remained at the workplace. The transition to working from home was implicated as a key determinant of the unhealthy changes in behavior [10, 34], but the cross-sectional analysis employed in these studies limit firm conclusions. In contrast, results from our longitudinal analysis indicated no significant changes in physical activity or sedentary time among those who started working from home in response to the pandemic. This finding is also consistent with another longitudinal study of Swiss office workers that reported no change in physical activity or sedentary time before (January 2020) and after the pandemic (April 2020) [14]. That said, adults in our study who were *able to start* working from home during the pandemic were among the most sedentary (11.0 hrs/d) and least active group (5.1 hrs/d total activity) in the population, comparable to much older retired or disabled adults in our sample. The high levels of sedentary time and lower activity appear to be due to underlying differences in occupational sitting and physical activity in these two occupational groups. Thus, working from home may not lead to more sedentary time and less activity, but rather, adults who can work from home may be more likely to have these less healthy behavioral characteristics. Adults in this group were more educated and more likely to be employed in more sedentary white collar service jobs, such as business and finance, and the educational and health sectors [9].

Ultimately, it is unclear what proportion of working adults will continue to work from home after the pandemic resolves, but our finding that this group reported sitting about 1.5 hrs/d longer than the national average in 2019 [16] raises concern since this amount of sedentary time has been associated with increased risk for mortality from cardiovascular disease or all causes [35]. Additionally, people who report no longer engaging in recommended levels of physical activity during the pandemic also report worse mental health characteristics [36] and physical inactivity has been associated with increased risk for severe COVID19 outcomes [37]. Regular moderate-vigorous intensity activity has also been associated with better immune function, reduced risk of community acquired infection, and lower infectious disease mortality [38], suggesting the need to include physical activity expertise in planning for future pandemics. Further efforts to reduce sedentary time and to increase physical activity among adults engaged in more sedentary occupations (e.g., [39, 40]) appear warranted. Adults working from home, for example, might be encouraged to exchange their time saved commuting for a regular routine of moderate-to-vigorous intensity physical activity. There are a number of proven strategies to help increase levels of physical activity that individuals or small groups can use, for example setting and tracking goals with a step counter [29]. Furthermore, neighborhood and home-based activities such as active play with children, dog-walking, or cycling/walking with friends can help foster activity despite lingering concerns over the pandemic.

This study has both strengths and limitations. An important limitation of our study is that we did not have data during the early period of the pandemic. While we cannot describe changes in each period of the pandemic the consistent strong reductions in the early days of the pandemic in the US [2–4] our findings of changes only in population subgroups by Fall 2020 suggest that overall, PA levels have largely returned to prepandemic levels. Furthermore, to complete a true longitudinal analysis, we were unable to oversample or expand our data collection in 2020 to focus changes within more vulnerable population groups. Use of self-reported information derived from ACT24 is both a strength and a limitation. ACT24 may be limited in capturing physical activity intensity of time-use data with the precision of device-based measures, but previous-day recall instruments [17, 41] have been shown to provide accurate estimates of physical activity and sedentary behavior for populations of individuals. The ability of ACT24 to capture contextual details about a variety of behaviors including domain-specific time use [18, 22] is also a strength of the method. Observed increases in time in-bed/sleeping for adults aged 40–64 are of interest, however, ACT24 does not assess sleep duration directly, thus our findings for time spent in-bed/sleeping should be interpreted cautiously. Further, ACT24 was designed to capture only primary activities and is somewhat limited in assessing specific childcare activities which limited our ability to investigate changes in activity associated with childcare, which often occurs as a secondary activity in the home, as well as other secondary activities in more detail. Further strengths of our study include its use of a nationwide sample of US adults in the AmeriSpeak panel who were selected to be representative of the US population and we conducted our surveys in the Fall of each year to minimize the impact of seasonal variation in behavior. We also employed a longitudinal study design with a relatively high follow-up response rate (~70%) for panel participants over a 12-month period. Overall consistency of our time-use results with the ATUS suggest the present results are a useful description of changes in time use in US adults [9].

## Conclusion

During the Fall of 2020, US adults on average reported spending less time in overall transportation and more total time in leisure pursuits, with little or no increase in exercise and recreational activities compared to pre-pandemic levels. Both men and women appeared to use the extra discretionary leisure-time in sedentary rather than in physically active behaviors. Adults with younger children reported the highest increases in sedentary time and reductions in moderate-to-vigorous intensity activity. Furthermore, adults who started working from home did not report changes in their activity levels but were found to be among the most sedentary and least active subgroups in the population, both before and during the pandemic. These findings point to the need for enhanced support for adults with young families to help them maintain their activity levels and highlight the unique challenges for adults who continue to or start working from home because of the COVID-19 pandemic. The prevalence of working from home is unlikely to return to pre-pandemic levels; therefore, implementing evidence-based individual and community-based approaches to increase physical activity and reduce sedentary time [42] may be needed to prevent this trend from adversely affecting health.

## Supporting information

S1 Fig Mean values for daily behaviors in 2020, by occupational status and location. (DOCX)

S1 Table ACT24 participant site detail: Major categories and selectable behaviors. (DOCX)

S2 Table Time-use classifications derived from ACT24 major category reports. ^1^Major categories separated by semi-colon. ^2^Other category is composed of Categories or data occurrences with a low prevalence of reporting.
(DOCX)

S3 Table Demographic characteristics (%) of participants reporting data in 2019 and 2020 (n = 1,635) comparing unweighted and weighted samples to the current population survey (CPS)^1^. CPS 2020 = US Census Current Population Survey, March 2020. ^1^Values are for participants prior to ACT24 quality control procedures. ^2^Difference = difference in proportions between Weighted and CPS 2020 estimates.
(DOCX)

S4 Table Difference (Δ) in time spent in daily behaviors pre- (2019) and mid-pandemic (2020), by race and ethnicity. ^1^Additional groups include, individuals self-identifying as non-Hispanic Asian, Other, or being from more than one race or ethnic group.
(DOCX)

S5 Table Difference (Δ) in time spent in daily behaviors pre- (2019) and mid-pandemic (2020), by the age of children in the household-US adults. (DOCX)

S6 Table Difference (Δ) in time spent in time-use pre- (2019) and mid-pandemic (2020), by the age of children in the household-US adults. (DOCX)

S1 Data Analytic data for this study (SAS dataset). (SAS7BDAT)
