# Supplementary material for: Changes in physical activity and sedentary time in United States adults in response to COVID-19
Source: PLoS One. 2022 Sep 9;17(9):e0273919. doi: 10.1371/journal.pone.0273919 (PMC9462823; doi:10.1371/journal.pone.0273919)
Supplement: S1 Fig — (DOCX) [file pone.0273919.s001.docx]

**S1 Figure.** Mean values for daily behaviors in 2020, by occupational status and location (longitudinal sample).
