# Supplementary material for: Changes in physical activity and sedentary time in United States adults in response to COVID-19
Source: PLoS One. 2022 Sep 9;17(9):e0273919. doi: 10.1371/journal.pone.0273919 (PMC9462823; doi:10.1371/journal.pone.0273919)
Supplement: S1 Table — (DOCX) [file pone.0273919.s002.docx]

**S1 Tables.** ACT24 Participant Site detail: Major Categories and selectable behaviors

| **Major Categories** | **Behaviors Available for Selection in each Category** |
| --- | --- |
|  |  |
| **Sleeping or napping** |  |
|  | Sleeping or in bed |
|  | Taking a nap |
| **Personal care** |  |
|  | Eating a meal or snack |
|  | Dressing or undressing |
|  | Grooming activities |
|  | Going to the bathroom |
|  | Showering or bathing |
|  | Other |
| **Housework** |  |
|  | Food preparation and serving |
|  | Wash dishes or clean-up kitchen |
|  | Clean or straighten-up house |
|  | Clean floors (vacuum, mop, sweep) |
|  | Unload and put away groceries |
|  | Laundry, put clothes away |
|  | Pet care (feed, let outside, groom) |
|  | Walking the dog |
|  | Pay bills, finance (desk work) |
|  | Sweeping outside: sidewalk, garage, or deck |
|  | Move heavy household items |
|  | Other |
| **Transportation commuting or travel** |  |
|  | Driving or riding in a car or truck |
|  | Motorcycle or motor scooter |
|  | Public transport (bus, train) |
|  | Waiting for bus, train, or a ride |
|  | Walking for transportation |
|  | Bicycling for transportation |
|  | Other |
| **Television, internet, digital media** |  |
|  | Watching TV, videos, movies |
|  | Email, texting, instant messaging |
|  | Browsing internet and social networks |
|  | Playing computer or electronic games |
|  | Other |
|  |  |
|  |  |
|  |  |
|  |  |
|  |  |
| **Communication, leisure, social activities** |  |
|  | Talking or socializing (in-person) |
|  | Talking with others (phone or computer) |
|  | Reading (books, papers, magazines) |
|  | Playing computer or electronic games |
|  | Playing games |
|  | Sitting quietly |
|  | Arts, crafts, hobbies |
|  | Eating out |
|  | Go to the movies |
|  | Attending sporting or cultural events |
|  | Other |
| **Shopping, errands, and appointments** |  |
|  | Walking (to and from stores) |
|  | Food shopping |
|  | Other shopping (not for food) |
|  | Waiting (in line for others) |
|  | Online shopping |
|  | Medical or dental appointments |
|  | Personal services |
|  | Other errands |
|  | Other |
| **Caring for or playing with others** |  |
|  | Childcare (dress, bath, feed) |
|  | Sitting or reclining with child |
|  | Elder care (dress, bath, feed) |
|  | Pet care (feed, let outside, groom) |
|  | Watching children play |
|  | Play with children (sit or stand) |
|  | Play with children (walk or run) |
|  | Play with animals (sit or stand) |
|  | Play with animals (walk or run) |
|  | Other |
| **Occupation, working for pay** |  |
|  | Desk or computer work |
|  | Attend meeting a meeting or presentation |
|  | Driving or riding in a vehicle |
|  | Eating and socializing at work |
|  | Sitting: other work |
|  | Standing: other work |
|  | Standing: some walking in work area |
|  | Standing: much walking in work area |
|  | Walking: longer distance |
|  | Carrying or moving heavy objects |
|  | Hard manual labor |
|  | Other |
| **Exercise, sports, active recreation** |  |
|  | Walking for exercise |
|  | Bicycling or exercise bike |
|  | Cardio machines |
|  | Running or jogging |
|  | Stretching or flexibility exercises |
|  | Weightlifting |
|  | Calisthenics |
|  | Aerobic dance |
|  | Water aerobics |
|  | Golf - practice |
|  | Golf - riding in cart |
|  | Golf - walking |
|  | Racquet sports |
|  | Swimming - recreational |
|  | Swimming - laps |
|  | Backpacking |
|  | Badminton |
|  | Basketball |
|  | Bowling |
|  | Boxing, punching bag, sparring |
|  | Canoeing or rowing a boat |
|  | Coaching a team |
|  | Dancing - slow |
|  | Dancing - fast |
|  | Downhill skiing (only active periods) |
|  | Fishing |
|  | Football, playing catch |
|  | Football, game play |
|  | Frisbee, playing catch |
|  | Frisbee, ultimate |
|  | Hiking |
|  | Handball |
|  | Horseback riding |
|  | Hunting |
|  | Ice hockey |
|  | Ice skating |
|  | Jumping rope |
|  | Martial arts (judo, karate, etc.) |
|  | Paddleball |
|  | Rock climbing |
|  | Skating, roller or in-line skates |
|  | Rowing for exercise |
|  | Rugby |
|  | Sailing |
|  | Skateboarding |
|  | Snorkeling |
|  | Scuba diving |
|  | Sledding |
|  | Snow shoeing |
|  | Soccer |
|  | Softball / baseball |
|  | Surfing |
|  | Table tennis, ping pong |
|  | Tai chi |
|  | Volleyball |
|  | Water skiing (only active periods) |
|  | Wrestling |
|  | Yoga |
|  | Other |
| **Lawn and garden** |  |
|  | Mow lawn (riding mower) |
|  | Mow lawn (push mower) |
|  | Use weed eater, blower or edger |
|  | Pick up lawn (debris, toys, tools) |
|  | Weed flower beds or garden |
|  | Water lawn or garden |
|  | Pick vegetables, fruit, or flowers |
|  | Trim haul branches |
|  | Sweep sidewalk, garage, or deck |
|  | Rake leaves or grass |
|  | Digging or shoveling |
|  | Stack or chop wood |
|  | Snow removal: using shovel |
|  | Snow removal: using broom |
|  | Snow removal: using blower |
|  | Other |
|  |  |
|  |  |
|  |  |
|  |  |
| **Home and auto maintenance or repair** |  |
|  | Interior remodeling |
|  | Interior decorating |
|  | Carry heavy items |
|  | Exterior work on house |
|  | Wash car |
|  | Auto repair |
|  | Sweep sidewalk, garage, or deck |
|  | Snow removal: using shovel |
|  | Snow removal: using broom |
|  | Snow removal: using blower |
|  | Other |
| **Church or spiritual pursuits** |  |
|  | Attend service |
|  | Sunday school |
|  | Meditating |
|  | Praying |
|  | Attend meetings |
|  | Caring for children |
|  | Sing, play choir |
|  | Socializing with others |
|  | Food preparation, serving, clean up |
|  | Cleaning, repair, maintenance |
|  | Other |
| **Volunteer work** |  |
|  | Desk or computer work |
|  | Attend meeting a meeting or presentation |
|  | Driving or riding in a vehicle |
|  | Eating and socializing while volunteering |
|  | Sitting: other work |
|  | Standing: other work |
|  | Standing: some walking in work area |
|  | Standing: much walking in work area |
|  | Walking: longer distance |
|  | Carrying or moving heavy objects |
|  | Hard manual labor |
|  | Other |
| **School and education** |  |
|  | Attending class |
|  | Attending laboratory sessions |
|  | Walk between classes |
|  | Studying: reading, taking notes, etc. |
|  | Giving presentation |
|  | Other activities |
|  |  |
