# Supplementary material for: Changes in physical activity and sedentary time in United States adults in response to COVID-19
Source: PLoS One. 2022 Sep 9;17(9):e0273919. doi: 10.1371/journal.pone.0273919 (PMC9462823; doi:10.1371/journal.pone.0273919)
Supplement: S2 Table — 1Major categories separated by semi-colon. 2Other category is composed of Categories or data occurrences with a low prevalence of reporting. (DOCX) [file pone.0273919.s003.docx]

| **S2 Tables.** Time-use classifications derived from ACT24 Major Category reports | |
| --- | --- |
|  |  |
| **Time-use/Life Domain Classifications** | **ACT24 Major Categories Used to Classify^1^** |
| **Personal** | Personal care |
| **Leisure Time** | Television, internet, digital media; Communication, leisure, social activities; Exercise, sports, active recreation |
| **Work** | Occupation, Working for Pay |
| **Household** | Housework; Shopping, errands and appointments; Caring for or playing with others; Lawn and garden; Home and auto maintenance or repair |
| **Transport** | Transportation, commuting or travel |
| **Other^2^** | Church or spiritual pursuits; Volunteer; School/education; Private time; Unreported (gap) time |
|  |  |
| ^1^Major categories separated by semi-colon | |
| **^2^**Other category is composed of Categories or data occurrences with a low prevalence of reporting | |
