# Supplementary material for: Changes in physical activity and sedentary time in United States adults in response to COVID-19
Source: PLoS One. 2022 Sep 9;17(9):e0273919. doi: 10.1371/journal.pone.0273919 (PMC9462823; doi:10.1371/journal.pone.0273919)
Supplement: S3 Table — CPS 2020 = US Census Current Population Survey, March 2020. 1Values are for participants prior to ACT24 quality control procedures. 2Difference = difference in proportions between Weighted and CPS 2020 estimates. (DOCX) [file pone.0273919.s004.docx]

| **S3 Tables.**  Demographic characteristics (%) of participants reporting data in 2019 and 2020 (n=1,635) comparing unweighted and weighted samples to the Current Population Survey (CPS)^1^ | | | | |
| --- | --- | --- | --- | --- |
|  | | |  |  |
| **Demographic Characteristics** | **Unweighted** | **Weighted** | **CPS 2020** | **Difference^2^** |
| **Sex** |  |  |  |  |
| Male | 56 | 49 | 49 | 0 |
| Female | 45 | 51 | 51 | 0 |
|  |  |  |  |  |
| **Age (years)** |  |  |  |  |
| 20-34 | 31 | 31 | 30 | 1 |
| 35-49 | 31 | 26 | 28 | -2 |
| 50-65 | 27 | 28 | 28 | 0 |
| 65-75 | 12 | 15 | 15 | 0 |
|  |  |  |  |  |
| **Race and ethnicity** |  |  |  |  |
| White, non-Hispanic | 71 | 62 | 62 | 0 |
| Black, non-Hispanic | 10 | 12 | 12 | 0 |
| Hispanic | 11 | 17 | 17 | 0 |
| Asian/Pacific Islander | 4 | 5 | 7 | -2 |
| Additional groups, non-Hispanic | 4 | 4 | 2 | 2 |
|  |  |  |  |  |
| **Educational attainment** |  |  |  |  |
| Less than High School | 2 | 4 | 8 | -4 |
| High School Equivalent | 12 | 31 | 27 | 4 |
| Some college | 37 | 28 | 28 | 0 |
| Bachelor's/Graduate degree | 50 | 37 | 37 | 0 |
|  |  |  |  |  |
| **Marital Status** |  |  |  |  |
| Currently married | 55 | 51 | 55 | -4 |
| Separated/Divorced/Single/Other | 45 | 49 | 45 | 4 |
|  |  |  |  |  |
| **Household Income** |  |  |  |  |
| Less than $30,000 | 17 | 22 | 14 | 9 |
| $30,000 to $74, 999 | 39 | 41 | 30 | 11 |
| $75,000 to $124, 999 | 27 | 24 | 25 | -1 |
| $125,000 or more | 17 | 14 | 32 | -18 |
|  |  |  |  |  |
| **Household Ownership** |  |  |  |  |
| Owner Occupied | 66 | 65 | 69 | -4 |
| Renting/Other | 34 | 35 | 31 | 4 |
| CPS 2020 = US Census Current Population Survey, March 2020 | |  |  |  |
| ^1^Values are for participants prior to ACT24 quality control procedures  ^2^Difference = difference in proportions between Weighted and CPS 2020 estimates | | |  |  |
