# Supplementary material for: Changes in physical activity and sedentary time in United States adults in response to COVID-19
Source: PLoS One. 2022 Sep 9;17(9):e0273919. doi: 10.1371/journal.pone.0273919 (PMC9462823; doi:10.1371/journal.pone.0273919)
Supplement: S4 Table — 1Additional groups include, individuals self-identifying as non-Hispanic Asian, Other, or being from more than one race or ethnic group. (DOCX) [file pone.0273919.s005.docx]

**S4 Tables.**  Difference (**Δ**) in time spent in daily behaviors pre- (2019) and mid-pandemic (2020), by race and ethnicity

|  | **White, non-Hispanic**  (n=1,171) | | | **Black, non-Hispanic**  (n=146) | | | **Hispanic**  (n=181) | | | **Additional groups^1^**  (n=137) | | |
| --- | --- | --- | --- | --- | --- | --- | --- | --- | --- | --- | --- | --- |
|  | Mean 2019 | **Δ** | p | Mean 2019 | **Δ** | p | Mean 2019 | **Δ** | p | Mean 2019 | **Δ** | p |
| **Daily behaviors** |  |  |  |  |  |  |  |  |  |  |  |  |
| In-bed/sleep | 8.19 | 0.09 | 0.33 | **7.19** | **0.82** | **0.05** | 8.29 | -0.14 | 0.67 | 7.87 | 0.17 | 0.64 |
| Sedentary | 9.76 | 0.09 | 0.63 | 9.84 | -0.36 | 0.60 | 9.08 | 0.33 | 0.48 | 10.16 | 0.21 | 0.76 |
| Total Physical Activity | 6.06 | -0.18 | 0.31 | 6.97 | -0.46 | 0.55 | 6.64 | -0.20 | 0.68 | 5.97 | -0.37 | 0.46 |
| Light | 3.71 | -0.06 | 0.69 | 4.29 | 0.07 | 0.93 | 4.43 | -0.17 | 0.64 | 3.83 | 0.19 | 0.65 |
| Moderate-vigorous | 2.35 | -0.12 | 0.43 | 2.67 | -0.53 | 0.32 | 2.21 | -0.03 | 0.94 | 2.13 | -0.56 | 0.16 |
| **Total time-use** |  |  |  |  |  |  |  |  |  |  |  |  |
| Leisure | **5.77** | **0.42** | **0.03** | 5.13 | -0.12 | 0.83 | 4.84 | 0.72 | 0.13 | 4.90 | 0.31 | 0.57 |
| Work | 3.38 | 0.01 | 0.95 | 4.51 | -0.19 | 0.78 | 3.96 | -0.17 | 0.76 | 4.42 | -0.28 | 0.65 |
| House | 3.05 | 0.04 | 0.76 | 2.54 | -0.30 | 0.44 | 2.98 | -0.25 | 0.45 | 2.85 | -0.16 | 0.67 |
| Transport | **1.18** | **-0.41** | **<.01** | **1.55** | **-0.70** | **0.00** | **1.00** | **-0.45** | **0.00** | **1.25** | **-0.62** | **0.00** |
| Personal Care | 1.80 | -0.07 | 0.27 | 1.57 | 0.40 | 0.14 | 1.82 | 0.00 | 0.98 | 1.96 | 0.01 | 0.97 |
| Other | 0.65 | -0.09 | 0.20 | 1.52 | 0.09 | 0.90 | 1.11 | 0.29 | 0.38 | 0.75 | 0.57 | 0.29 |
| **Sedentary time-use** |  |  |  |  |  |  |  |  |  |  |  |  |
| Leisure | 4.85 | **0.45** | **0.01** | 3.84 | 0.14 | 0.76 | 3.90 | 0.85 | 0.08 | 4.04 | 0.21 | 0.71 |
| Work | 1.77 | 0.14 | 0.34 | 2.24 | 0.07 | 0.89 | 1.79 | 0.10 | 0.78 | 2.70 | -0.14 | 0.75 |
| House | 0.48 | -0.02 | 0.64 | 0.48 | -0.02 | 0.93 | 0.58 | **-0.22** | **0.03** | 0.47 | 0.07 | 0.71 |
| Transport | 1.12 | **-0.39** | **<.01** | 1.46 | **-0.66** | **0.00** | 0.94 | **-0.41** | **0.00** | 1.16 | **-0.56** | **0.00** |
| Personal Care | 1.01 | -0.02 | 0.66 | 0.73 | 0.45 | 0.08 | 0.92 | -0.12 | 0.34 | 1.13 | 0.22 | 0.54 |
| Other | 0.53 | -0.07 | 0.29 | 1.10 | -0.33 | 0.20 | 0.95 | 0.14 | 0.58 | 0.66 | 0.42 | 0.43 |
| **Active time-use** |  |  |  |  |  |  |  |  |  |  |  |  |
| Leisure | 0.92 | -0.03 | 0.73 | 1.29 | -0.25 | 0.37 | 0.94 | -0.13 | 0.45 | 0.86 | 0.10 | 0.52 |
| Work | 1.61 | -0.13 | 0.43 | 2.27 | -0.26 | 0.63 | 2.17 | -0.27 | 0.53 | 1.72 | -0.13 | 0.77 |
| House | 2.56 | 0.06 | 0.60 | 2.06 | -0.28 | 0.37 | 2.40 | -0.03 | 0.91 | 2.39 | -0.22 | 0.51 |
| Transport | 0.05 | -0.02 | 0.06 | 0.09 | -0.03 | 0.33 | 0.06 | **-0.04** | **0.03** | 0.08 | **-0.07** | **0.00** |
| Personal Care | 0.79 | -0.05 | 0.19 | 0.84 | -0.05 | 0.68 | 0.89 | 0.12 | 0.37 | 0.84 | **-0.20** | **0.04** |
| Other | 0.12 | -0.02 | 0.39 | 0.42 | 0.42 | 0.52 | 0.17 | 0.15 | 0.32 | 0.09 | 0.15 | 0.11 |

^1^Additional groups include, individuals self-identifying as non-Hispanic Asian, Other, or being from more than one race or ethnic group
