# Supplementary material for: Changes in physical activity and sedentary time in United States adults in response to COVID-19
Source: PLoS One. 2022 Sep 9;17(9):e0273919. doi: 10.1371/journal.pone.0273919 (PMC9462823; doi:10.1371/journal.pone.0273919)
Supplement: S5 Table — (DOCX) [file pone.0273919.s006.docx]

**S5 Tables.** Difference (**Δ**) in time spent in daily behaviors pre- (2019) and mid-pandemic (2020), by the age of children in the household-US adults

|  |  | **≤ 5 yrs**  (n=176) | | | | **6-12 yrs**  **(**n=306) | | | | **13-17 yrs**  (n=231) | | | **No children < 18 yrs**  (n=1,138) | | | |
| --- | --- | --- | --- | --- | --- | --- | --- | --- | --- | --- | --- | --- | --- | --- | --- | --- |
| **Overall sample** | **Metric** | **Mean 2019** | **Δ** | **p** | **Mean 2019** | | **Δ** | **p** | **Mean 2019** | | **Δ** | **p** | **Mean**  **2019** | **Δ** | **P** |  |
|  | Bed/sleep | 8.29 | -0.27 | 0.32 | 8.06 | | 0.20 | 0.47 | 8.09 | | 0.19 | 0.47 | 8.04 | 0.12 | 0.18 |  |
|  | Sedentary | **8.21** | **0.82** | **0.01** | **8.61** | | **0.60** | **0.05** | 8.62 | | 0.21 | 0.64 | 10.13 | 0.02 | 0.93 |  |
|  | Total PA | 7.51 | -0.55 | 0.10 | **7.33** | | **-0.79** | **0.03** | 7.29 | | -0.39 | 0.40 | 5.83 | -0.14 | 0.39 |  |
|  | Light | 4.50 | 0.17 | 0.58 | 4.27 | | 0.05 | 0.87 | 4.23 | | 0.30 | 0.44 | 3.77 | -0.08 | 0.48 |  |
|  | Mod-vigorous | **3.02** | **-0.72** | **0.02** | **3.07** | | **-0.84** | **0.03** | 3.06 | | -0.69 | 0.09 | 2.06 | -0.06 | 0.67 |  |
| **Men** |  |  |  |  |  |  |  |  |  |  |  |  |  |  |  |  |
|  | Bed/sleep | 8.07 | -0.06 | 0.81 | 7.92 | | 0.25 | 0.34 | 7.99 | | 0.17 | 0.53 | 7.81 | 0.09 | 0.41 |  |
|  | Sedentary | **8.58** | **0.97** | **0.02** | **9.09** | | **1.05** | **0.03** | 8.51 | | 0.00 | 1.00 | 10.59 | -0.09 | 0.77 |  |
|  | Total PA | **7.35** | **-0.89** | **0.03** | **6.98** | | **-1.27** | **0.00** | 7.50 | | -0.16 | 0.74 | 5.61 | 0.00 | 0.99 |  |
|  | Light | 3.75 | -0.26 | 0.49 | 3.60 | | -0.55 | 0.08 | 3.80 | | 0.26 | 0.66 | 3.28 | 0.13 | 0.57 |  |
|  | Mod-vigorous | 3.60 | -0.63 | 0.17 | 3.38 | | -0.72 | 0.10 | 3.70 | | -0.43 | 0.46 | 2.33 | -0.13 | 0.44 |  |
| **Women** |  |  |  |  |  | |  |  |  | |  |  |  |  |  |  |
|  | Bed/sleep | 8.43 | -0.41 | 0.41 | 8.17 | | 0.16 | 0.73 | 8.18 | | 0.19 | 0.66 | 8.29 | 0.16 | 0.35 |  |
|  | Sedentary | 7.96 | 0.76 | 0.14 | 8.26 | | 0.40 | 0.44 | 8.72 | | 0.38 | 0.57 | 9.65 | 0.12 | 0.58 |  |
|  | Total PA | 7.62 | -0.35 | 0.53 | 7.58 | | -0.56 | 0.35 | 7.11 | | -0.59 | 0.40 | 6.07 | -0.28 | 0.09 |  |
|  | Light | 4.98 | 0.39 | 0.44 | 4.74 | | 0.32 | 0.49 | 4.62 | | 0.33 | 0.55 | 4.29 | -0.29 | 0.07 |  |
|  | Mod-vigorous | **2.64** | **-0.74** | **0.02** | 2.85 | | -0.88 | 0.06 | **2.49** | | **-0.91** | **0.04** | 1.78 | 0.01 | 0.94 |  |
