# Supplementary material for: Changes in physical activity and sedentary time in United States adults in response to COVID-19
Source: PLoS One. 2022 Sep 9;17(9):e0273919. doi: 10.1371/journal.pone.0273919 (PMC9462823; doi:10.1371/journal.pone.0273919)
Supplement: S6 Table — (DOCX) [file pone.0273919.s007.docx]

**S6 Tables.** Difference (**Δ**) in time spent in time-use pre- (2019) and mid-pandemic (2020), by the age of children in the household-US adults

|  |  | **< 5 yrs**  (n=176) | | |  | **6-12 yrs**  **(**n=306) | | |  | **13-17 yrs**  (n=231) | | |  | **No children < 18 yrs**  (n=1,138) | | | |
| --- | --- | --- | --- | --- | --- | --- | --- | --- | --- | --- | --- | --- | --- | --- | --- | --- | --- |
| **Overall**  **Sample** | **Domain** | **Mean 2019** | **Δ** | **p** |  | **Mean 2019** | **Δ** | **p** |  | **Mean 2019** | **Δ** | **p** |  | **Mean 2019** | **Δ** | **p** |  |
|  | **Total time-use** |  |  |  |  |  |  |  |  |  |  |  |  |  |  |  |  |
|  | Leisure | 3.74 | 0.37 | 0.33 |  | 4.30 | 0.64 | 0.05 |  | 4.67 | 0.86 | 0.04 |  | 5.96 | 0.33 | 0.06 |  |
|  | Work | 4.02 | -0.08 | 0.85 |  | 4.28 | 0.01 | 0.98 |  | 4.46 | -0.39 | 0.40 |  | 3.44 | -0.07 | 0.67 |  |
|  | House | 4.41 | 0.18 | 0.56 |  | 3.43 | -0.21 | 0.48 |  | 2.83 | -0.18 | 0.57 |  | 2.78 | -0.05 | 0.56 |  |
|  | Transport | **1.31** | **-0.58** | **0.00** |  | **1.19** | **-0.48** | **0.00** |  | **1.24** | **-0.50** | **0.01** |  | **1.16** | **-0.45** | **<.01** |  |
|  | Personal Care | 1.58 | 0.17 | 0.36 |  | 1.79 | -0.08 | 0.62 |  | 1.58 | 0.17 | 0.24 |  | 1.84 | 0.00 | 0.97 |  |
|  | Other | 0.66 | 0.22 | 0.20 |  | 0.94 | -0.08 | 0.71 |  | 1.13 | -0.15 | 0.60 |  | 0.79 | 0.12 | 0.38 |  |
|  | **Sedentary time-use** | | | | | | | | | | | | | | | | |
|  | Leisure | 2.78 | 0.35 | 0.17 |  | 3.44 | 0.71 | 0.06 |  | 3.58 | 1.04 | 0.02 |  | 4.99 | 0.38 | 0.03 |  |
|  | Work | 1.84 | 0.62 | 0.09 |  | 1.71 | 0.44 | 0.07 |  | 1.50 | 0.10 | 0.70 |  | 1.98 | 0.00 | 0.98 |  |
|  | House | 0.99 | 0.21 | 0.19 |  | 0.68 | 0.01 | 0.89 |  | 0.64 | -0.25 | 0.03 |  | 0.40 | -0.06 | 0.35 |  |
|  | Transport | **1.27** | **-0.55** | **0.00** |  | **1.14** | **-0.45** | **0.00** |  | **1.19** | **-0.45** | **0.01** |  | **1.10** | **-0.42** | **<.01** |  |
|  | Personal Care | 0.81 | 0.05 | 0.65 |  | 0.97 | -0.11 | 0.40 |  | 0.84 | -0.14 | 0.30 |  | 1.01 | 0.10 | 0.20 |  |
|  | Other | 0.52 | 0.14 | 0.45 |  | 0.66 | 0.00 | 1.00 |  | 0.87 | -0.09 | 0.67 |  | 0.65 | 0.02 | 0.87 |  |
|  | **Active time-use** | | | | | | | | | | | | | | | | |
|  | Leisure | 0.96 | 0.02 | 0.93 |  | 0.86 | -0.07 | 0.64 |  | 1.08 | -0.18 | 0.36 |  | 0.97 | -0.05 | 0.38 |  |
|  | Work | **2.18** | **-0.70** | **0.01** |  | 2.57 | -0.43 | 0.20 |  | 2.95 | -0.49 | 0.23 |  | 1.45 | -0.07 | 0.61 |  |
|  | House | 3.42 | -0.03 | 0.91 |  | 2.75 | -0.22 | 0.33 |  | 2.20 | 0.07 | 0.80 |  | 2.37 | 0.00 | 0.97 |  |
|  | Transport | **0.04** | **-0.04** | **0.00** |  | **0.05** | **-0.03** | **0.05** |  | **0.06** | **-0.04** | **0.02** |  | **0.06** | **-0.03** | **0.00** |  |
|  | Personal Care | 0.77 | 0.12 | 0.46 |  | 0.83 | 0.04 | 0.75 |  | **0.74** | **0.32** | **0.04** |  | **0.83** | **-0.09** | **0.00** |  |
|  | Other | 0.14 | 0.08 | 0.53 |  | 0.28 | -0.08 | 0.51 |  | 0.26 | -0.06 | 0.63 |  | 0.14 | 0.11 | 0.33 |  |
|  |  | | | | | | | | | | | | | | | | |
| **Men** | **Total time-use** | | | | | | | | | | | | | | | | |
|  | Leisure | 3.73 | -0.11 | 0.79 |  | 4.33 | 0.41 | 0.16 |  | 4.52 | 0.63 | 0.22 |  | **6.37** | **0.45** | **0.05** |  |
|  | Work | 5.73 | 0.37 | 0.49 |  | 5.19 | 0.62 | 0.18 |  | 5.65 | -0.48 | 0.38 |  | 3.74 | 0.01 | 0.97 |  |
|  | House | 3.15 | -0.37 | 0.27 |  | **2.74** | **-0.74** | **0.02** |  | 1.90 | 0.03 | 0.92 |  | 2.35 | -0.18 | 0.18 |  |
|  | Transport | 1.24 | -0.31 | 0.24 |  | **1.42** | **-0.56** | **0.02** |  | **1.53** | **-0.66** | **0.05** |  | **1.16** | **-0.48** | **<.01** |  |
|  | Personal Care | 1.44 | 0.47 | 0.12 |  | 1.77 | 0.03 | 0.91 |  | 1.38 | 0.41 | 0.07 |  | 1.81 | -0.09 | 0.21 |  |
|  | Other | 0.64 | 0.03 | 0.86 |  | 0.62 | 0.02 | 0.95 |  | 1.02 | -0.11 | 0.70 |  | 0.77 | 0.21 | 0.29 |  |
|  | **Sedentary time-use** | | | | | | | | | | | | | | | | |
|  | Leisure | 2.67 | 0.25 | 0.32 |  | 3.54 | 0.48 | 0.06 |  | 3.49 | 0.69 | 0.11 |  | 5.38 | 0.38 | 0.13 |  |
|  | Work | 2.63 | 0.83 | 0.07 |  | **2.19** | **1.14** | **0.01** |  | 1.64 | 0.24 | 0.43 |  | 2.03 | 0.11 | 0.45 |  |
|  | House | 0.72 | -0.06 | 0.71 |  | 0.51 | -0.04 | 0.79 |  | 0.37 | -0.16 | 0.06 |  | 0.36 | -0.10 | 0.09 |  |
|  | Transport | 1.19 | -0.26 | 0.32 |  | **1.35** | **-0.52** | **0.03** |  | 1.43 | -0.58 | 0.07 |  | **1.09** | **-0.44** | **<.01** |  |
|  | Personal Care | 0.77 | 0.17 | 0.30 |  | 1.02 | -0.03 | 0.87 |  | 0.70 | 0.07 | 0.40 |  | 1.06 | -0.01 | 0.84 |  |
|  | Other | 0.59 | 0.03 | 0.87 |  | 0.48 | 0.02 | 0.95 |  | 0.88 | -0.25 | 0.25 |  | 0.68 | -0.02 | 0.82 |  |
|  | **Active time-use** | | | | | | | | | | | | | | | | |
|  | Leisure | 1.06 | -0.36 | 0.28 |  | 0.79 | -0.07 | 0.67 |  | 1.04 | -0.05 | 0.89 |  | 0.99 | 0.07 | 0.57 |  |
|  | Work | 3.10 | -0.46 | 0.17 |  | 2.99 | -0.52 | 0.21 |  | 4.01 | -0.71 | 0.19 |  | 1.72 | -0.10 | 0.64 |  |
|  | House | 2.43 | -0.31 | 0.18 |  | **2.23** | **-0.70** | **0.00** |  | 1.53 | 0.19 | 0.48 |  | 1.99 | -0.08 | 0.48 |  |
|  | Transport | **0.05** | **-0.05** | **0.00** |  | 0.07 | -0.04 | 0.14 |  | **0.09** | **-0.08** | **0.05** |  | 0.07 | -0.03 | 0.07 |  |
|  | Personal Care | 0.66 | 0.30 | 0.30 |  | 0.75 | 0.06 | 0.79 |  | 0.68 | 0.34 | 0.08 |  | **0.75** | **-0.08** | **0.03** |  |
|  | Other | 0.05 | 0.00 | 0.97 |  | 0.14 | 0.00 | 1.00 |  | 0.14 | 0.15 | 0.39 |  | 0.09 | 0.23 | 0.22 |  |
|  |  | | | | | | | | | | | | | | | | |
| **Women** | **Total time-use** | | | | | | | | | | | | | | | | |
|  | Leisure | 3.74 | 0.66 | 0.15 |  | 4.28 | 0.78 | 0.10 |  | 4.79 | 1.05 | 0.06 |  | 5.53 | 0.19 | 0.40 |  |
|  | Work | 2.90 | -0.27 | 0.65 |  | 3.64 | -0.24 | 0.63 |  | 3.39 | -0.30 | 0.61 |  | 3.12 | -0.18 | 0.35 |  |
|  | House | 5.23 | 0.45 | 0.31 |  | 3.93 | 0.02 | 0.97 |  | 3.67 | -0.38 | 0.40 |  | 3.22 | 0.11 | 0.44 |  |
|  | Transport | **1.36** | **-0.75** | **0.00** |  | **1.02** | **-0.40** | **0.01** |  | 0.99 | -0.35 | 0.06 |  | **1.16** | **-0.43** | **<.01** |  |
|  | Personal Care | 1.68 | -0.02 | 0.93 |  | 1.81 | -0.14 | 0.41 |  | 1.76 | -0.04 | 0.83 |  | 1.87 | 0.11 | 0.40 |  |
|  | Other | 0.68 | 0.33 | 0.29 |  | 1.17 | -0.17 | 0.60 |  | 1.23 | -0.20 | 0.66 |  | 0.81 | 0.03 | 0.87 |  |
|  | **Sedentary time-use** | | | | | | | | | | | | | | | | |
|  | Leisure | 2.85 | 0.40 | 0.29 |  | 3.37 | 0.85 | 0.13 |  | 3.67 | 1.35 | 0.04 |  | 4.57 | 0.37 | 0.06 |  |
|  | Work | 1.32 | 0.53 | 0.32 |  | 1.37 | 0.09 | 0.71 |  | 1.38 | -0.02 | 0.95 |  | 1.94 | -0.12 | 0.47 |  |
|  | House | 1.16 | 0.36 | 0.11 |  | 0.81 | 0.02 | 0.87 |  | 0.88 | -0.33 | 0.07 |  | 0.45 | -0.01 | 0.94 |  |
|  | Transport | **1.32** | **-0.72** | **0.00** |  | **0.99** | **-0.38** | **0.01** |  | 0.97 | -0.33 | 0.07 |  | **1.11** | **-0.40** | **0.00** |  |
|  | Personal Care | 0.83 | -0.02 | 0.87 |  | 0.93 | -0.16 | 0.30 |  | 0.96 | -0.33 | 0.13 |  | 0.95 | 0.21 | 0.11 |  |
|  | Other | 0.48 | 0.21 | 0.45 |  | 0.79 | -0.03 | 0.87 |  | 0.86 | 0.05 | 0.87 |  | 0.63 | 0.06 | 0.73 |  |
|  | **Active time-use** | | | | | | | | | | | | | | | | |
|  | Leisure | 0.89 | 0.26 | 0.33 |  | 0.91 | -0.08 | 0.72 |  | 1.12 | -0.30 | 0.20 |  | **0.95** | **-0.18** | **0.01** |  |
|  | Work | **1.58** | **-0.79** | **0.01** |  | 2.27 | -0.33 | 0.43 |  | 2.01 | -0.28 | 0.55 |  | 1.18 | -0.06 | 0.70 |  |
|  | House | 4.06 | 0.09 | 0.80 |  | 3.12 | -0.01 | 0.98 |  | 2.79 | -0.05 | 0.91 |  | 2.78 | 0.11 | 0.44 |  |
|  | Transport | **0.04** | **-0.03** | **0.04** |  | 0.03 | -0.02 | 0.11 |  | 0.02 | -0.01 | 0.12 |  | **0.06** | **-0.03** | **0.01** |  |
|  | Personal Care | 0.85 | 0.01 | 0.97 |  | 0.88 | 0.02 | 0.90 |  | 0.80 | 0.29 | 0.16 |  | **0.92** | **-0.10** | **0.03** |  |
|  | Other | 0.20 | 0.12 | 0.55 |  | 0.37 | -0.14 | 0.45 |  | 0.37 | -0.25 | 0.15 |  | 0.18 | -0.03 | 0.65 |  |
